# Viruses may facilitate the cyanobacterial blooming during summer bloom succession in Xiangxi Bay of Three Gorges Reservoir, China

**DOI:** 10.3389/fmicb.2023.1112590

**Published:** 2023-03-08

**Authors:** Kaida Peng, Yiying Jiao, Jian Gao, Wen Xiong, Yijun Zhao, Shao Yang, Mingjun Liao

**Affiliations:** ^1^School of Life Sciences, Central China Normal University, Wuhan, Hubei, China; ^2^Hubei Key Laboratory of Ecological Restoration for River-Lakes and Algal Utilization, School of Civil and Environmental Engineering, Hubei University of Technology, Wuhan, Hubei, China

**Keywords:** viruses, lysis, lysogeny, succession, cyanobacterial bloom, Three Gorges Reservoir

## Abstract

The occurrence of cyanobacterial blooms in summer are frequently accompanied by the succession of phytoplankton communities in freshwater. However, little is known regarding the roles of viruses in the succession, such as in huge reservoirs. Here, we investigated the viral infection characteristics of phytoplankton and bacterioplankton during the summer bloom succession in Xiangxi Bay of Three Gorges Reservoir, China. The results indicated that three distinct bloom stages and two successions were observed. From cyanobacteria and diatom codominance to cyanobacteria dominance, the first succession involved different phyla and led to a *Microcystis* bloom. From *Microcystis* dominance to *Microcystis* and *Anabaena* codominance, the second succession was different Cyanophyta genera and resulted in the persistence of cyanobacterial bloom. The structural equation model (SEM) showed that the virus had positive influence on the phytoplankton community. Through the Spearman’s correlation and redundancy analysis (RDA), we speculated that both the increase of viral lysis in the eukaryotic community and the increase of lysogeny in cyanobacteria may contributed to the first succession and *Microcystis* blooms. In addition, the nutrients supplied by the lysis of bacterioplankton might benefit the second succession of different cyanobacterial genera and sustain the dominance of cyanobacteria. Based on hierarchical partitioning method, the viral variables still have a marked effect on the dynamics of phytoplankton community, although the environmental attributes were the major factors. Our findings suggested that viruses played multiple potential roles in summer bloom succession and may help the blooms success of cyanobacteria in Xiangxi Bay. Under the background of increasingly serious cyanobacterial blooms worldwide, our study may have great ecological and environmental significance for understanding the population succession in phytoplankton and controlling the cyanobacterial blooms.

## 1. Introduction

Cyanobacterial blooms have increased in frequency around the world in recent decades (Huisman et al., 2018) and are likely to become more severe as a consequence of eutrophication, rising CO_2_ levels and accelerating global warming (Verspagen et al., 2014; Chapra et al., 2017). Moreover, cyanobacterial blooms can cause major problems, such as toxin production, hypoxia generation, and food web disruption, leading to the loss of ecosystem services (Verspagen et al., 2014; Chapra et al., 2017). However, some mechanisms underlying the ecological success of cyanobacteria remain unclear, which makes it difficult to deal with cyanobacterial blooms (Wilhelm et al., 2020).

In summer, cyanobacterial communities occur widely in freshwater by displacing eukaryotic algae, which generally includes diatom and green algae (Ke et al., 2008; Zepernick et al., 2021). In addition, the succession of phytoplankton among different cyanobacterial genera also occurs frequently (Moustaka-Gouni et al., 2006; Chun et al., 2020; Tanvir et al., 2021). Various factors, including nutrients, predation, temperature, light, pH, antibiotics, and water turbulence, have been found to influence these successions and the ecological success of cyanobacteria (Steffen et al., 2015; Reavie et al., 2016; Huisman et al., 2018; Wang et al., 2021; Xu et al., 2021; Zepernick et al., 2021). Given the complexity of cyanobacterial blooms, the drivers of cyanobacterial dominance and succession are still being explored (Wang et al., 2021).

Cases in past decades have shown that viruses are becoming more pronounced in phytoplankton community regulation (Suttle et al., 1990; Fuhrman, 1999; Brussaard, 2004; Knowles et al., 2016; Pound et al., 2020). As suggested by negative frequency-dependent selection, the “Kill-the-Winner” (KtW) model of lytic infection predicts that abundant prokaryotic types will be exposed to strong viral pressure for maintaining high prokaryotic richness (Winter et al., 2010). Many studies on phytoplankton blooms showed direct viral control and provided empirical support for the KtW model (Bratbak et al., 1993; Hewson et al., 2001; Baudoux et al., 2006). Under a modified KtW model, Pound et al. (2020) found that viruses may suppress the competition of eukaryotic community and allow for the cyanobacterial bloom. Recently, infection strategy of the “Piggyback-the-Winner” (PtW) model has been proposed in which lysogeny predominates at high microbial abundance and growth rates (Silveira and Rohwer, 2016). Several studies also supported the PtW model (Knowles et al., 2016; Coutinho et al., 2017). However, these different viral infection strategies could be favored depending on environmental conditions (Bongiorni et al., 2005; Payet and Suttle, 2013). Viral lysis and lysogenic infection also contribute to bacterioplankton community. Similar to phytoplankton, high viral lysis pressure will apply to dominant and fast-growing bacteria, which has been confirmed to have a major impact on bacterial diversity and the community structure, i.e., the “Kill-the-Winner” model (Winter et al., 2010). Besides, lysogeny also has been previously hypothesized to be a preferable survival strategy for both the virus and bacterioplankton (Paul, 2008). Notably, viral lysis predominantly channels particulate organic carbon and nutrients away from higher trophic levels, which was called “viral shunt” (Wilhelm and Suttle, 1999). Nutrients released by viral lysis of heterotrophic bacteria can be efficiently remineralized and transferred to phytoplankton (Weinbauer et al., 2011; Shelford and Suttle, 2018). Currently, the accumulated scientific evidence about the role of viruses is growing fast, but the information about the roles of viruses in bloom succession is still limited.

Cyanobacterial bloom succession has occurred frequently in several tributaries of the Three Gorges Reservoir (TGR), the world’s largest hydroelectric power project, since the initial water impounding in 2003 (Zhou et al., 2019). And the summer phytoplankton population succession in Xiangxi Bay of TGR in 2010 was in order as follows: diatom, green algae, cyanobacteria, and the main factors of were water temperature, water stability, and mixed layer depth (Fang et al., 2013). It also has been revealed that nutrients, temperature, light, and hydrodynamic regimes are the key environmental factors affecting the outbreaking and succession of blooms (Fang et al., 2013; Yang et al., 2022). As for viruses in reservoirs, Kopylov and Zabotkina (2021) investigated the distribution of viruses, the frequency of visibly infected cells of heterotrophic bacteria and autotrophic picocyanobacteria and their virus-induced mortality in six reservoirs along the Volga. The viral infection rate of picocyanobacteria in mesoeutrophic reservoir was higher than that in mesotrophic reservoir (Kopylov et al., 2010). Although a large number of reservoirs have been built worldwide, there have been very few studies on the ecological effects of viruses in these manipulated aquatic ecosystems in-depth, especially when the bloom occurs.

In the present study, we investigated the viral lysis and lysogeny of phytoplankton and bacterioplankton in the Xiangxi Bay (the largest tributary of the TGR, China) during a bloom succession event from July 18 to August 29. The abiotic environmental factors were also monitored to compare how they differ from the effects of viral factors during bloom succession. The results revealed that viruses played multiple potential roles in summer bloom succession in Xiangxi Bay of TGR and may facilitate the bloom success of cyanobacteria. These findings provide a foundation for further understanding phytoplankton succession and controlling the cyanobacterial blooms.

## 2. Materials and methods

### 2.1. Sampling and physicochemical variables

**Figure 1:**
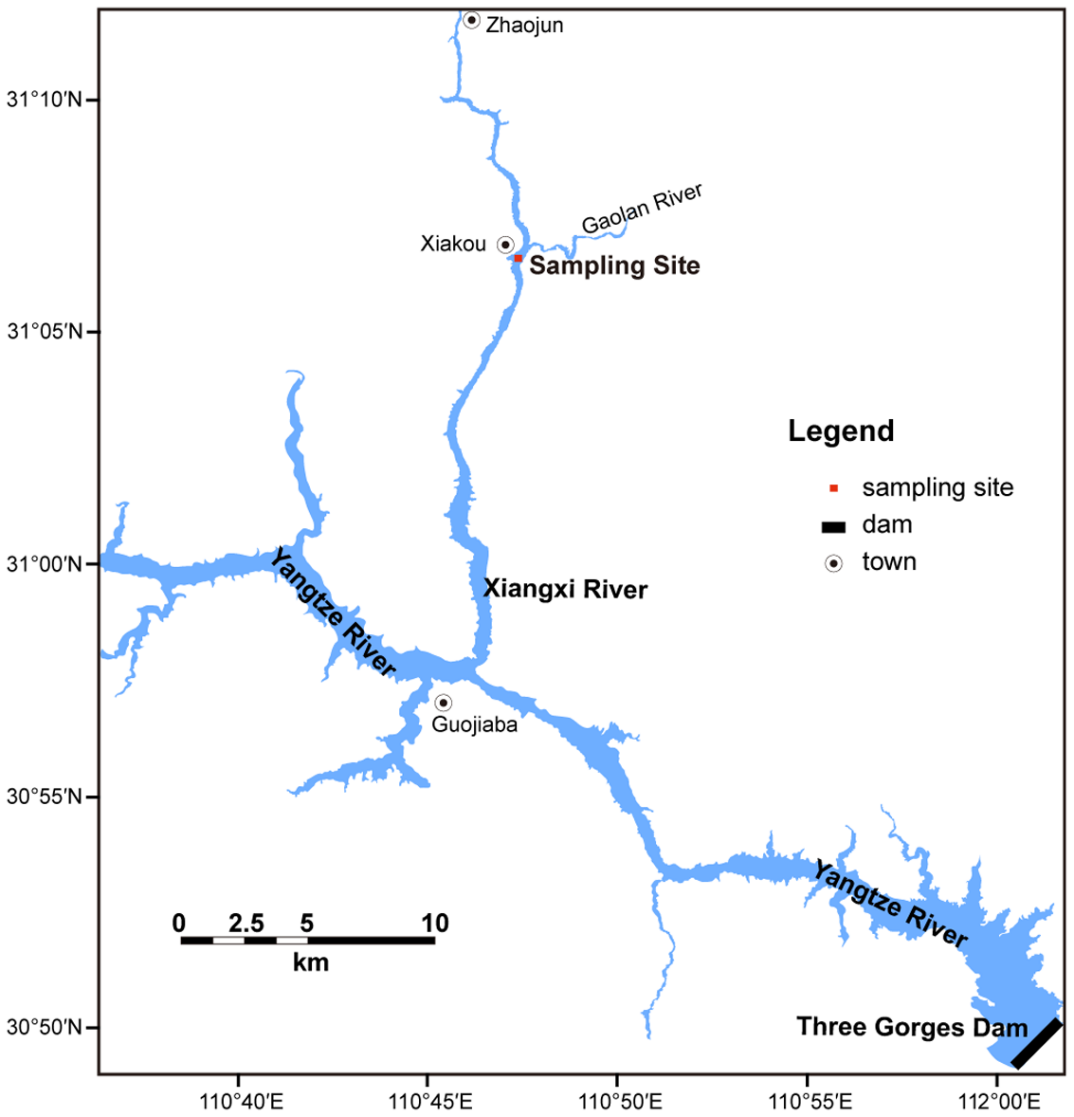
Location of the sampling site in Xiangxi Bay of Three Gorges Reservoir.

Xiangxi Bay is the largest tributary close to the Three Gorges Dam in Hubei Province, China. The main stream of Xiangxi Bay is 94 km with a basin area of 3,099 km^2^. The study site is located in the middle reaches of Xiangxi Bay (Figure 1), which is affected not only by the main stream of the TGR but also a typical bloom area (Yang et al., 2018).

To monitor viral and environmental parameter variations in summer blooms, water samples at 0.2 m below the water surface were collected at 10 am every 2 days from July 18th to August 29th, 2017. The total nitrogen (TN), total phosphorous (TP), PO_4_^3−^, permanganate index (COD_Mn_), dissolved silicate (D-Si), chlorophyll *a* (Chl-*a*), and water temperature (*T*) were determined as described by Nwankwegu et al. (2020). One liter of water samples were fixed with Lugol’s iodine solution (2% final concentration) and allowed to settle for 48 h. Phytoplankton species were identified according to Paerl et al. (2015). For the counting of microbial abundances, 5 mL of water samples were firstly fixed with 25% glutaraldehyde to a final concentration of 0.5% for 15–30 min at 4°C, then the samples were flash frozen and stored at −80°C until analysis. Ten liters water samples were collected and put into a polyethylene pot, and then the viral lysis and lysogenic fraction experiments were carried out within 1 h.

### 2.2. Microbial abundances

Viruses and bacterioplankton were counted simultaneously by epifluorescence microscopy (Steele et al., 2007). Briefly, 1 mL sub-samples from the glutaraldehyde-fixed samples vacuum-filtered onto an Anodisc 25 mm 0.02-μm filter (Whatman, Middlesex, United Kingdom) for epifluorescence microscopy. When necessary for accurate enumeration, samples were diluted with 0.02 μm-filtered water prior to filtration. The filter was stained for 15 min with SYBR green I solution (1:400) in the dark. After being dried, the filter was placed on a glass slide and mounted with an antifade mounting solution. For each filter, a minimum of 200 bacteria and 200 viruses were counted in random fields of view. Analyses were performed under 1,000 × magnification with an epifluorescence microscope (Leica DMR, Wetzlar, Germany) equipped with a 100 W high-pressure mercury lamp and using light filters for blue excitation (450–490 nm wide bandpass).

Phytoplankton was analyzed without staining, but by using their natural autofluorescence. Briefly, 2 ml water samples were filtered onto 0.22 μm pore size cellulose acetate layer sheets (Xinya, Shanghai, China). Then, phytoplankton were counted by their orange and red autofluorescence (that is, Chl *a*, present in all phytoplankton; Parésys et al., 2005) under blue excitation light (450–490 nm wide bandpass) using a Leica DMR microscope. To obtain reliable estimates of abundance of phytoplankton, at least 200 phytoplankton were counted in random fields per sample under a 200 × magnification.

### 2.3. Viral lysis rate

Phytoplankton community is controlled by viral induced lysis of phytoplankton directly and also influenced by the viral induced lysis of bacterioplankton indirectly (Weinbauer et al., 2011; Biggs et al., 2021). The modified dilution approach was used to determine the viral induced mortality on both phytoplankton and bacterioplankton (Parvathi et al., 2014; Tsai et al., 2015a). First, sampling water was gently passed through a 200 μm mesh, 0.2 μm membrane (Pall, Dreieich, Germany), and a 30 kDa tangential flow filtration system (Sartorius Stedim Biotech, Göttingen, Germany) to create mesoplankton-free whole water, grazer-free water, and virus-free water, respectively. Then, the mesoplankton-free whole water was mixed with 0.2 μm diluent or 30 kDa ultrafiltrate in proportions of 100, 70, 40, and 20%, to gradually decrease the mortality impact with increasing dilution. All experiments were performed in triplicate in 0.5 L clear polycarbonate bottles. After preparation of the two parallel dilution series, a 3 ml subsample was taken for phytoplankton and bacterioplankton enumeration as specified in section 2.2. And the bottles were incubated for 24 h *in situ*. After the 24-h incubation, a second phytoplankton and bacterioplankton count was executed. Apparent phytoplankton and bacterioplankton growth rates (μ, d^−1^) of 0.2 μm and 30 kDa diluent series (i.e., 100, 70, 40, and 20%) are calculated from the changes in abundance during the incubation using the equation:


$$\mu =\ln\;\left(P_{t}/P_{0}\right)/t$$

where *P_t_* and *P*_0_ are the final and initial measured phytoplankton and bacterioplankton abundance, respectively, and *t* is the duration of the experiment.

Linear regression analysis of apparent growth rates against fraction of water is applied to each of the dilution experimental series (0.2 μm and 30 kDa diluent series). The grazing rates of phytoplankton and bacterioplankton were estimated from the regression coefficient of the apparent growth rate for the 0.2 μm series, whereas the combined rate of viral induced lysis and grazing was estimated from the regression for the 30 kDa series. Viral mortality of phytoplankton and bacterioplankton were determined from the corresponding significant difference between the two regression coefficients of 0.2 μm and 30 kDa series (as tested by analysis of covariance; Kimmance et al., 2007).

### 2.4. The percent of lysogeny

Lysogeny in particular is assumed to be a beneficial life strategy for both hosts and viruses under unfavorable conditions (Weinbauer et al., 2003). The lysogenic fractions of phytoplankton and bacterioplankton were determined using the mitomycin C method (Williamson et al., 2002). One hundred milliliters of mesoplankton-free whole water (the sampling water filtered with 200 μm mesh) was filtered through a 0.2 μm filter (Pall, Dreieich, Germany) using a 47 mm filtration apparatus to reduce the volume to approximately 5 ml. Then, 100 ml of virus-free water (the sampling water filtered with 30 kDa membrane) was added back to the remaining 5 ml of the 0.2 μm filtered sample, and the volume was once again reduced to approximately 5 ml through filtration. The filtration and resuspension processes were repeated three times. Subsequently, virus-reduced samples were either added to a final concentration of 1 μg/mL mitomycin C (Sigma-Aldrich, St. Louis, United States) or left untreated as controls. All samples were incubated at room temperature in the dark for 24 h and counted using the method as described in section 2.2 to obtain the abundances of phytoplankton and bacterioplankton after 24 h. The frequency of lysogenic cells (FLC) of phytoplankton and bacterioplankton were calculated according to the following equation:


$$FLC=\frac{\left(C_{24}-T_{24}\right)}{C_{24}}\times 100\%$$

where, *C*_24_ and *T*_24_ are the number of phytoplankton or bacterioplankton enumerated in the control and induced samples at 24 h, respectively.

### 2.5. Statistical analysis

The taxonomic compositions of the phytoplankton communities were analyzed at the species level (for these with relative abundance >1%) and visualized using the R (v4.2.2) package “ggplot2”. To display the succession of phytoplankton community composition, hierarchical cluster analysis (HCA) and principal coordinate analysis (PCoA) were carried out using Bray–Curtis distance based on the relative abundance matrices of phytoplankton species. Permutational multivariate ANOVA (PERMANOVA, *n* = 999) was then used to examine the statistical significance of differences among the bloom stages (Anderson, 2001). According to the bloom stages defined by HCA, a box plot of viral lysis rate and the percent of lysogeny was constructed and one way ANOVA was performed to test the difference among the bloom stages.

To understand the relationship between viral factors and phytoplankton community succession, the structural equation modeling (SEM) was used to test the pathway that the virus changed the phytoplankton community. All variables were transformed by log10 (*x* + 1) before SEM and we determined the latent variables first. To support a conclusion that virus shaped the blooms succession (i.e., the change of phytoplankton community), the latent variable “Phytoplankton” was determined as a proxy for phytoplankton community change. As for other latent variables which have close relation with latent variable phytoplankton community, we concluded as “Virus”, “Nutrient”, and “Physical factor” in our study. We next chose the observed variables to each latent variable. Spearman’s correlation analysis was performed to examine the relationships among all variables, and variables were filtered with high correlation to simplify the modeling. Then, we took the most likely paths in consideration and checked the suitability of the estimated parameters. The SEM was finally established after the remove of some observed variables. And the SEM fitness was examined on the basis of a non-significant chi-square (*χ*^2^) test (*p* > 0.05), the comparative fit index (CFI > 0.95), and the root mean square error of approximation (RMSEA < 0.05; Jonsson and Wardle, 2010; Shen et al., 2019).

Spearman’s correlation analysis was also used to identify the correlations between the phytoplankton community and viral factors (including viral abundance, viral-induced lysis rate of phytoplankton, viral-induced lysis rate of bacterioplankton, frequency of lysogenic phytoplankton, and frequency of lysogenic bacterioplankton) using the R package “psych”. As for phytoplankton community, relevant indicators include abundance of different species of phytoplankton, Shannon Wiener index, PCo1 (principal component score in axis 1 of PCoA) and PCo2 (principal component score in axis 2 of PCoA). Only statistically significant correlations (*p* < 0.05) are shown. Due to the result of the longest gradient lengths obtained by detrended correspondence analysis (DCA) were <3, we further performed redundancy analysis (RDA) to examine the effect of environmental factors (including viral factors) on the succession of summer blooms using “vegan” package of the statistical language R. Hellinger transformation of phytoplankton abundance was carried out before performing RDA to minimize the effect of zeroes in the community data, and environmental factors were transformed with log10 (*x* + 1) to approximate a normal distribution. To obtain the parsimonious model, environmental variables were selected by calculating variance inflation factors, and environmental variables with variance inflation factors >8 were removed. Then, a Monte Carlo test (999 permutations) based on the RDA was used to assess the significance of RDA model and each selected variable (*p* < 0.05; Brener-Raffalli et al., 2018; Hao et al., 2020). And we further determined the explanation effect of each selected environmental factor on the RDA results based on the hierarchical partitioning method using the “rdacca.hp” package in R (Lai et al., 2022).

## 3. Results

### 3.1. Dynamics of bloom characteristics

**Figure 2:**
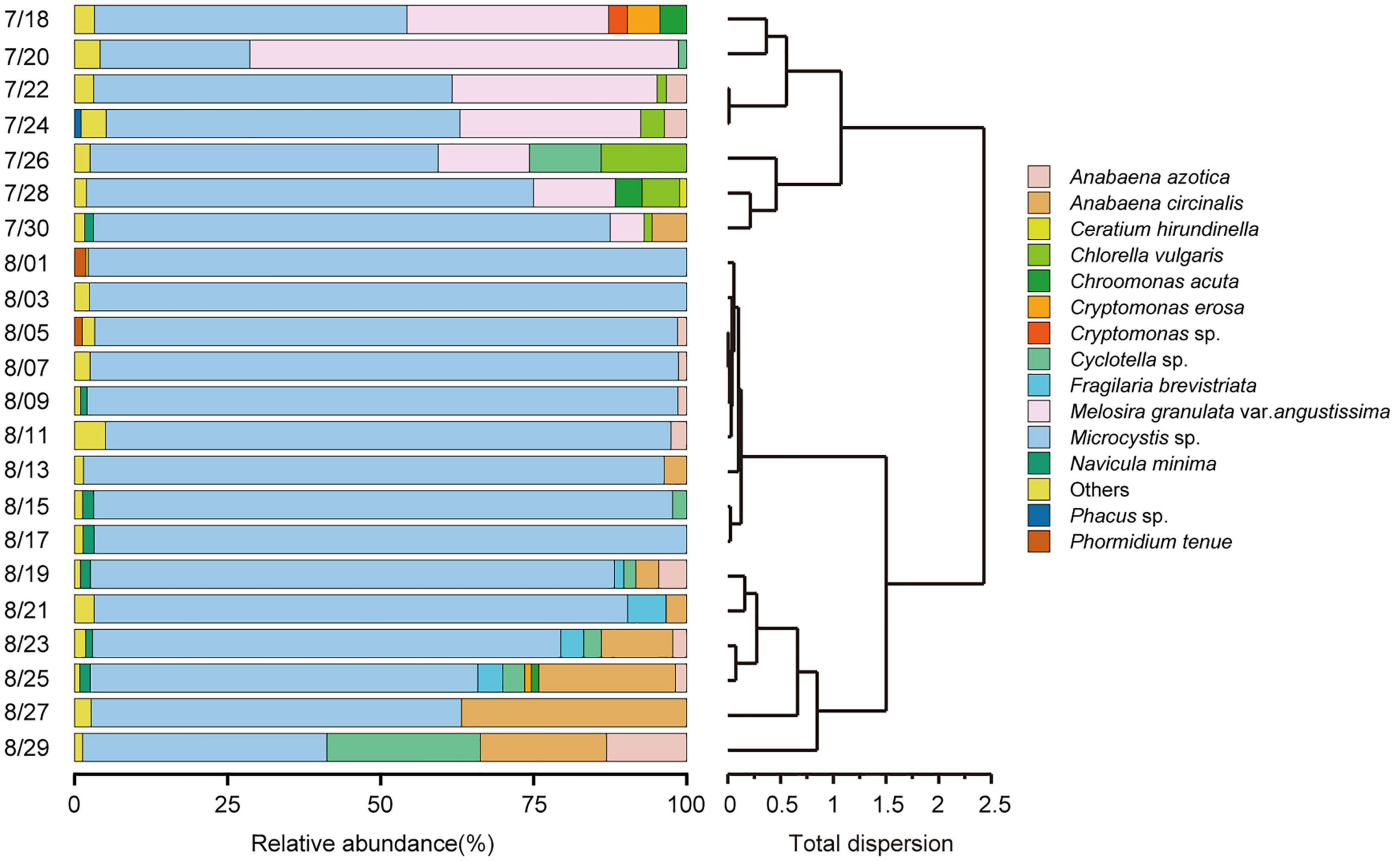
Temporal trends in phytoplankton community composition at the species level (relative abundance of >1%) and hierarchical cluster analysis (HCA) were applied to identify the succession of phytoplankton compositional change based on Bray–Curtis dissimilarity matrices.

**Figure 3:**
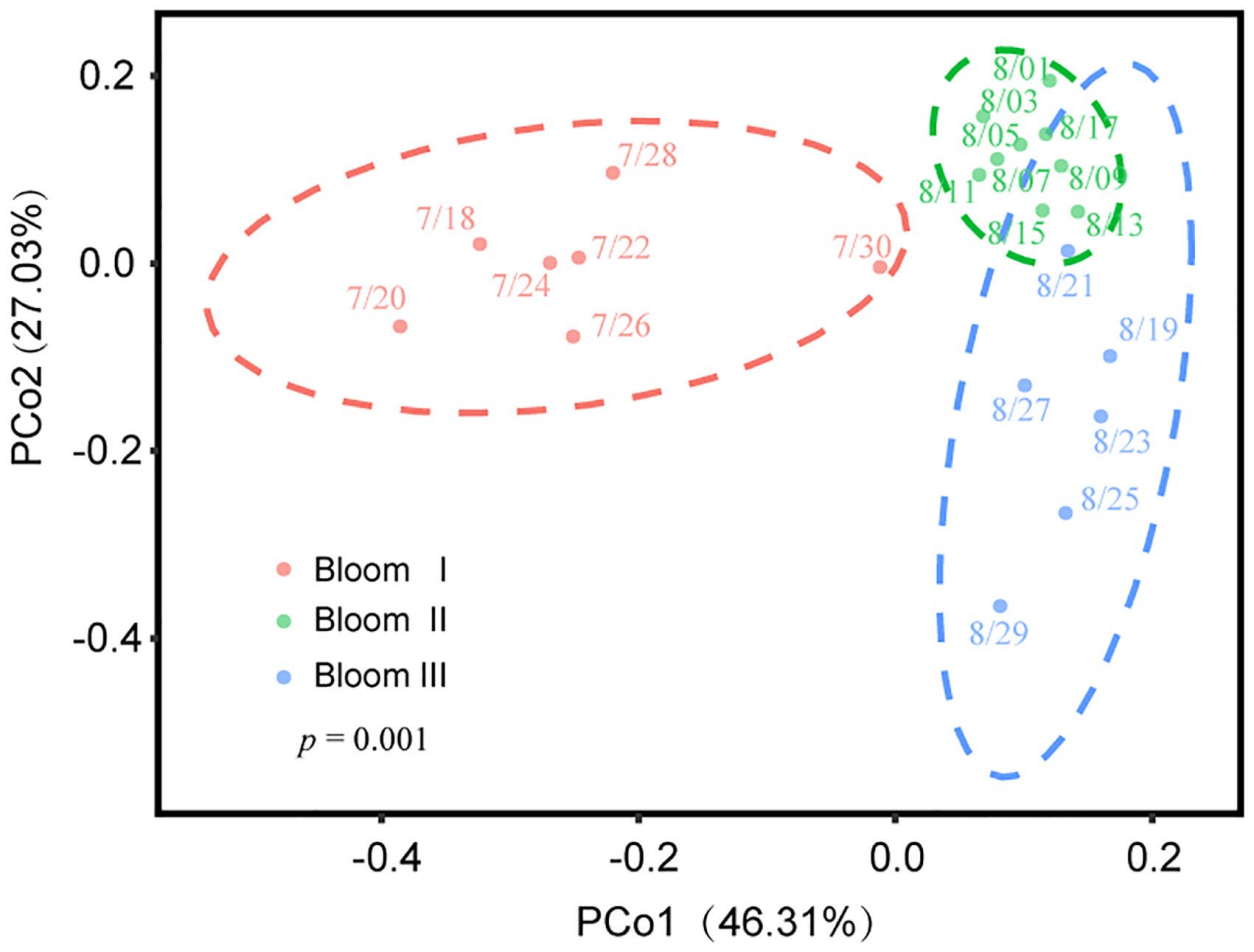
Variation in beta diversity visualized using principal coordinate analysis (PCoA). Different color regions represented the 95% bootstrapped confidence ellipses of each bloom stage. The PERMANOVA test determined that phytoplankton communities diverged significantly in these three stages (*p* = 0.001).

According to the HCA (Figure 2) and PCoA (Figure 3), three significant (*p* < 0.05) summer bloom stages were observed: Bloom I (18th July—30th July), Bloom II (30th July—17th August), and Bloom III (17th August—29th August). There was an increasing trend in the mean concentrations of Chl *a*, which were 23.27, 29.18, and 45.50 μg/L, respectively (Table 1). Both *Microcystis* sp. (Cyanophyta) and *Melosira granulata* var. *angustissima* (Bacillariophyta) were dominant in Bloom I. However, only *Microcystis* sp. dominated in Bloom II. *Microcystis* sp. and *Anabaena circinalis* (Cyanophyta) were the main species in Bloom III. Corresponding to the three stages, there were two successions, including the first succession from eukaryotic algae to cyanobacteria (Bloom I–Bloom II) and the second succession among different cyanobacterial genera (Bloom II–Bloom III).

**Table 1:** Mean (±SD) microbial and environmental characteristics of the summer bloom succession in Xiangxi Bay from 18th July to 29th August 2017.

	Bloom I mean ± SD	Bloom II mean ± SD	Bloom III mean ± SD
Viral abundance (10^7^ VLPs/mL)	0.29 ± 0.10	0.67 ± 0.79	0.71 ± 0.32
Bacterial abundance (10^6^ cells/mL)	1.90 ± 0.80	1.43 ± 0.47	1.45 ± 0.43
Phytoplankton abundance (10^6^ cells/L)	4.84 ± 2.42	26.62 ± 51.88	8.36 ± 6.56
Bacillariophyta (10^6^ cells/L)	1.73 ± 1.77	0.15 ± 0.13	0.58 ± 0.54
Dinoflagellata (10^6^ cells/L)	0.03 ± 0.04	0.02 ± 0.02	0.01 ± 0.01
Cyanophyta (10^6^ cells/L)	2.67 ± 1.18	26.41 ± 51.73	7.63 ± 6.01
Euglenophyta (10^6^ cells/L)	0.01 ± 0.01	0.00	0.00
Chlorophyta (10^6^ cells/L)	0.28 ± 0.44	0.03 ± 0.02	0.02 ± 0.02
Cryptophyta (10^6^ cells/L)	0.13 ± 0.13	0.02 ± 0.02	0.07 ± 0.07
TN (mg/L)	1.78 ± 0.24	1.77 ± 0.49	1.26 ± 0.53
TP (mg/L)	0.12 ± 0.14	0.11 ± 0.07	0.10 ± 0.03
COD_Mn_ (mg/L)	2.13 ± 0.22	2.09 ± 1.66	2.54 ± 0.41
PO_4_^3−^ (mg/L)	0.006 ± 0.003	0.001 ± 0.001	0.004 ± 0.001
D-Si (mg/L)	8.42 ± 5.66	4.93 ± 1.14	5.10 ± 0.25
Temperature (°C)	28.00 ± 0.50	28.52 ± 0.81	26.50 ± 0.37
Chl *a* (μg/L)	23.27 ± 9.47	29.18 ± 36.92	45.50 ± 21.44

VLPs, virus like particles; TN, total nitrogen; TP, total phosphorus; D-Si, dissolved silicate; and Chl *a*, chlorophyll *a*.

There was an increase in the abundance of viruses and a decrease of bacterioplankton along the bloom succession (Table 1). For the abiotic characteristics (Table 1), the TN concentrations were 1.78, 1.77, and 1.26 mg/L, respectively, which decreased markedly in Bloom III. The TP concentration decreased gradually with succession proceeded and was 0.12, 0.11, and 0.10 mg/L along the three bloom stages. The concentrations of PO_4_^3−^ and D-Si first decreased and then increased. In addition, the average water temperature first increased slightly and then decreased to 28.00, 28.52, and 26.50°C, respectively. Temporal variation of environmental characteristics in the summer bloom succession was shown in Supplementary Table S1.

### 3.2. Viral lysis and lysogeny

To assess influence of viruses on phytoplankton community, we quantified the viral lysis to the mortality of phytoplankton and bacterioplankton by conducting 22 modified dilution assays during the summer blooms (see Supplementary Table S2 for details). And prophage induction by mitomycin C was also applied to quantify the percent of lysogenic phytoplankton and bacterioplankton (see Supplementary Table S2 for details).

**Figure 4:**
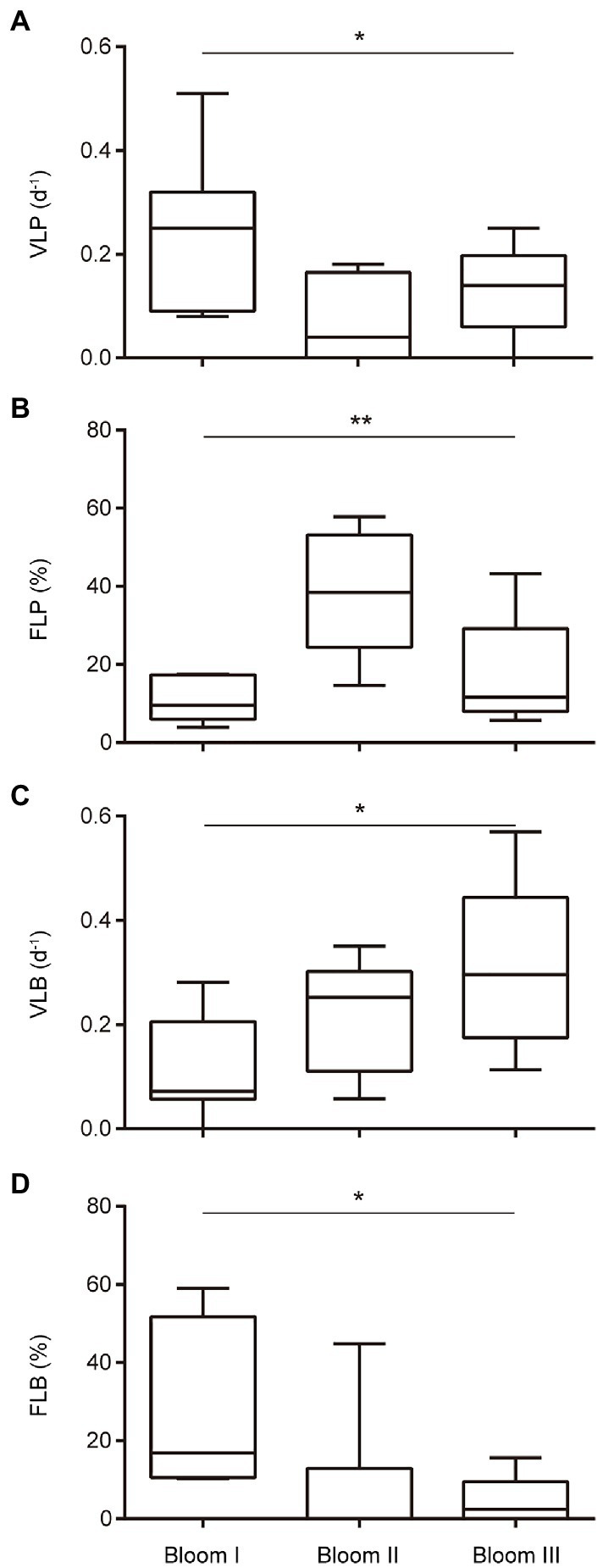
Boxplot of the viral-induced lysis rate of phytoplankton (VLP) **(A)**, viral-induced lysis rate of bacterioplankton (VLB) **(C)**, frequency of lysogenic phytoplankton (FLP) **(B)**, and frequency of lysogenic bacterioplankton (FLB) **(D)** in three bloom stages. ^*^ and ^**^ indicate significant differences among the three stages at *p* < 0.05 and *p* < 0.01, respectively.

The viral lysis rates of phytoplankton and bacterioplankton in the three bloom stages were significantly different (*p* < 0.05; Figure 4). The viral lysis rate of phytoplankton (VLP) in Bloom I, II, and III first decreased and then increased (Figure 4A). The mean VLP was the highest (0.251 d^−1^) in Bloom I and the lowest (0.124 d^−1^) in Bloom II. The viral lysis rate of bacterioplankton (VLB) increased gradually during the summer blooms and reached up to 0.312 d^−1^ in Bloom III (Figure 4C).

The frequency of lysogenic phytoplankton and bacterioplankton in the three bloom stages were also significantly different (*p* < 0.05; Figure 4). The frequency of lysogenic phytoplankton (FLP) first increased and then decreased, with the highest mean FLP (38.52%) in Bloom II (Figure 4B). The frequency of lysogenic bacterioplankton (FLB) were observed to decrease gradually from Bloom I to Bloom III (Figure 4D).

### 3.3. Effects of viruses on phytoplankton community succession

**Figure 5:**
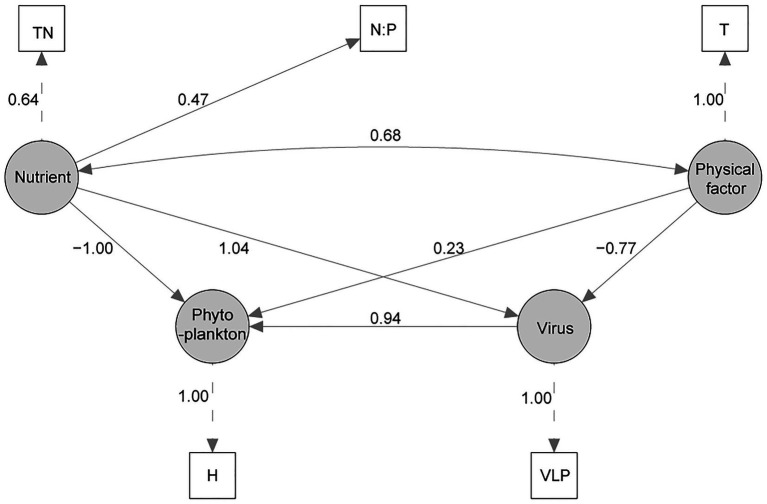
The structural equation model (SEM) of the effects of virus, nutrient, and physical variable on phytoplankton community. Measured variables are indicated with rectangles, latent (unmeasured) variables with ovals. Numbers beside each arrow indicate the standardized path coefficient. (TN, total nitrogen; N:P, TN/TP ratio; *T*, water temperature; VLP, viral-induced lysis rate of phytoplankton; H, Shannon Wiener index).

A structural equation model was successfully established (*χ*^2^ = 0.719, *p* = 0.698, CFI = 1.0, GFI = 0.987, RMSEA = 0; Figure 5). In the final SEM, Shannon Wiener index was used to model the latent variable “Phytoplankton” as a proxy for phytoplankton community change. The SEM analysis showed that Virus, Nutrient, and Physical factor all had influence on the phytoplankton diversity, and Virus and Nutrient had higher path coefficient than Physical factor. In latent variable “Virus”, VLP was the only important factor, and VLB, FLP, and FLB were deleted in SEM adjustments. Besides, TN and N:P were important to model the latent variable “Nutrient”, and PO_4_^3−^, COD_Mn_, D-Si were also deleted in SEM adjustments. Similarly, *T* was the only important factor to model the latent variable “Physical factor”.

**Figure 6:**
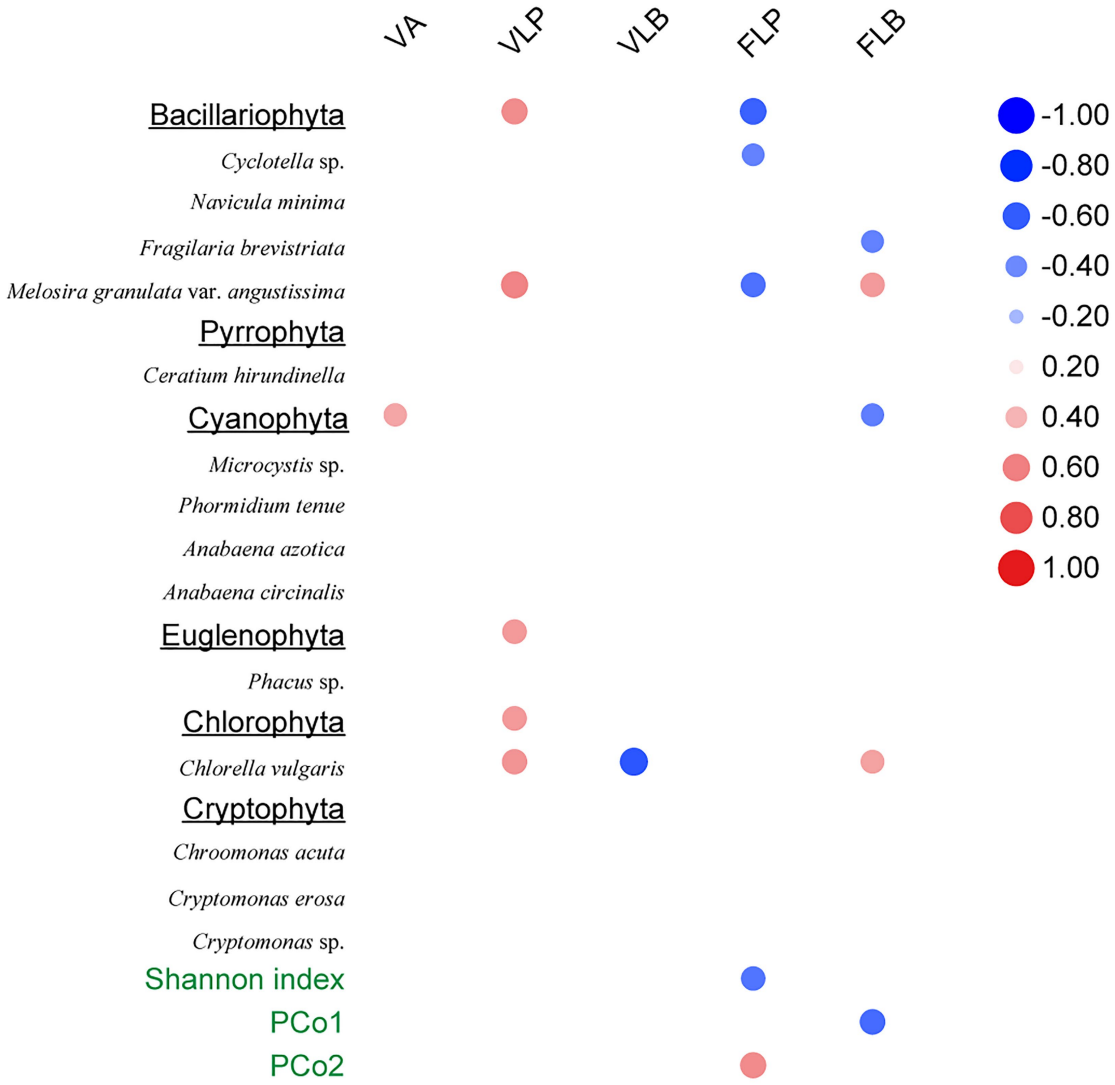
Spearman rank correlations between viral-related variables and the phytoplankton community composition. Only statistically significant (*p* < 0.05) correlations are shown. Right column data represent correlation coefficient.

The Spearman rank correlation analysis also showed strongly association between viral infection characteristics and phytoplankton community composition. Specifically, there was significant positive correlation between the VLP and Bacillariophyta, Euglenophyta as well as Chlorophyta (*p* < 0.05; Figure 6). PCoA results for the phytoplankton communities are shown in Figure 3. As the indexes of phytoplankton community structure, the first PCoA axis (PCo1) explained 46.31% of the total variance, and the second was 27.03%. And both the Shannon index of phytoplankton community and the values of axis 2 (PCo2) of PCoA exhibited significant correlations with the FLP (*p* < 0.05). The FLB had a significant negative correlation with the values of axis 1 (PCo1) of PCoA (*p* < 0.05).

**Figure 7:**
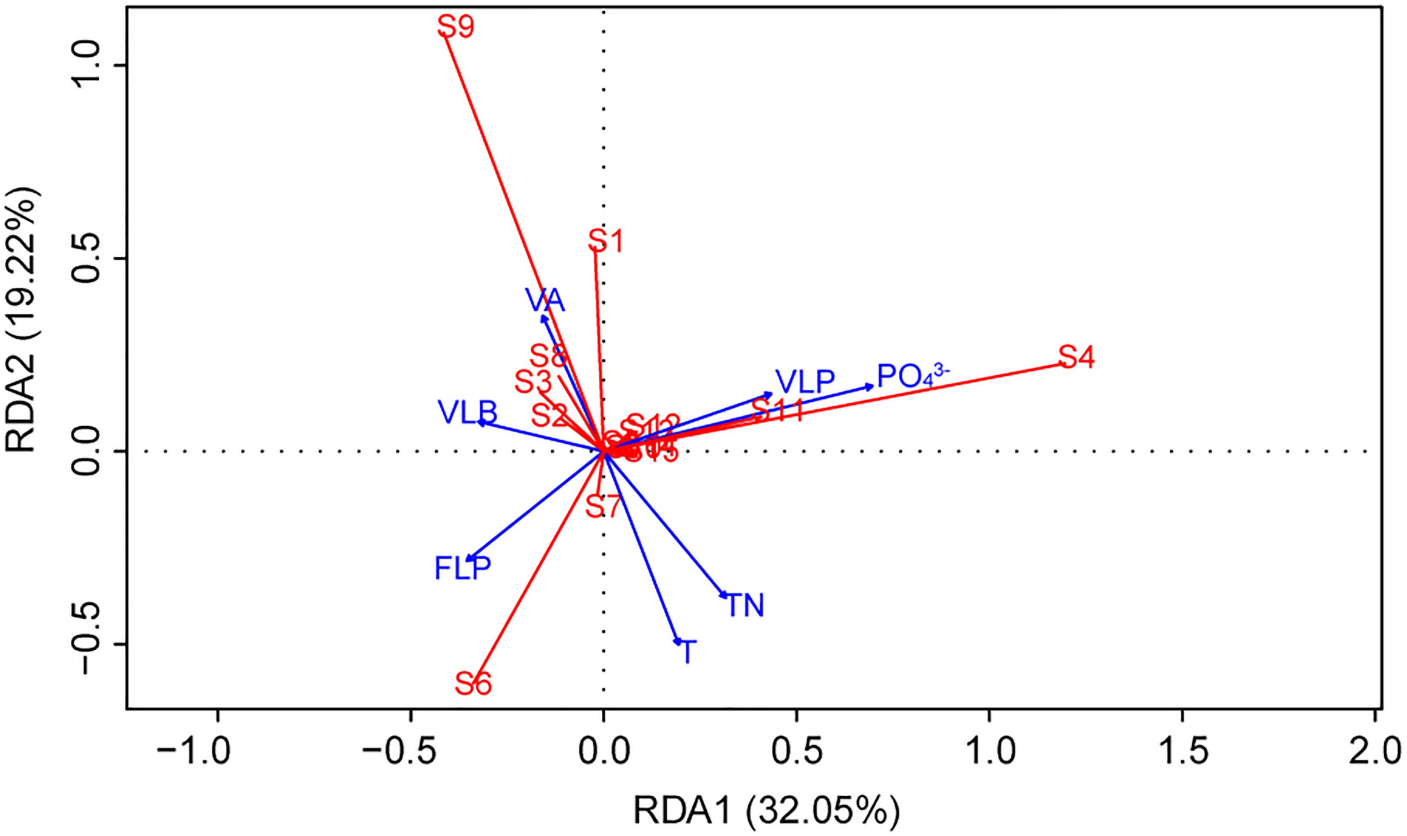
Redundancy analysis (RDA) ordination plot describing the phytoplankton community composition (response variables, in red) in relation to viral and environmental variables (explanatory variables, in blue). (S1, *Cyclotella* sp.; S2, *Navicula minima*; S3, *Fragilaria brevistriata*; S4, *Melosira granulata* var. *angustissima*; S5, *Ceratium hirundinella*; S6, *Microcystis* sp.; S7, *Phormidium tenue*; S8, *Anabaena azotica*; S9, *Anabaena circinalis*; S10, *Phacus* sp.; S11, *Chlorella vulgaris*; S12, *Chroomonas acuta*; S13, *Cryptomonas erosa*; S14, *Cryptomonas* sp.; and S15, Others).

Redundancy analysis was another method to explore the importance of viruses to the phytoplankton community. Monte Carlo test revealed that the RDA model was significant (*p* < 0.01), and also showed that the explanatory variables (TN, T, PO_4_^3−^, VA, VLP, VLB, and FLP) selected by variance inflation factors were contributed significantly (*p* < 0.05) to the RDA model (Table 2). The first two axes of the RDA (Figure 7) explained 32.05 and 19.22% of the variation in the data, respectively. This variation was closely related to the viral factors. For example, the eukaryotic algae, such as *M. granulata* var. *angustissima*, *Chlorella vulgaris*, *Chroomonas acuta*, *Cryptomonas erosa*, and *Cryptomonas* sp., were positively correlated with the VLP and were negatively correlated with the FLP (Figure 7). Besides, a positive relationship of the VLB, VA with *A. circinalis*, *Anabaena azotica*, and *Fragilaria brevistriata* were found (Figure 7). Hierarchical partitioning analysis demonstrated that viral factors (VA, VLP, VLB, and FLP) had obvious effect on the phytoplankton composition among the seven selected significant explanatory variables (Table 2) included in RDA model [total *R*^2^ (adj) = 0.433], which was lower than that independently explained by environmental factors (i.e., PO_4_^3−^, TN, and T).

**Table 2:** Percentage and significance of the seven selected explanatory variables to the summer phytoplankton community composition change.

Variables	Individual importance	I. Perc (%)^a^	*p* value^b^
TN	0.0580	13.39	0.0290
PO_4_^3−^	0.2097	48.43	0.0001
T	0.0724	16.72	0.0001
VA	0.0143	3.30	0.0229
VLP	0.0294	6.79	0.0447
VLB	0.0060	1.39	0.0491
FLP	0.0432	9.98	0.0191
Total	0.433	100	

a Individual effects as a proportion of total corrected *R*^2^.

b *p* value for the permutation test based on 999 randomizations.

## 4. Discussion

In the present study, three stages of summer bloom succession were observed (Figures 2, 3; Table 1) and this phenomenon occurs frequently in Xiangxi Bay of the TGR (Fang et al., 2013). Both phyto- and bacterioplankton lytic and lysogenic infection characteristics were significantly different in these three stages (Figure 4) and may have influenced the succession of summer blooms by different ways from the analysis of SEM, Spearman rank correlation, RDA, and Hierarchical partitioning (Table 2; Figures 5–7). And these effects of viruses in summer bloom succession may further have helped the success of cyanobacterial bloom.

To explore the causation between virus and the phytoplankton community succession, the biodiversity of Shannon Wiener index (H) was used as the observed variable of phytoplankton in SEM. The path coefficient between H and latent variable “phytoplankton” was 1.0, which showed that the SEM could explain the most of the variation of H. Nutrient, virus, and physical factor are the factors influence the H, but Nutrient and Virus are more important. Many other studies also have shown that nutrient was more important than climate change for phytoplankton community change (Yan et al., 2019; Zhang et al., 2021). As for virus, VLP was the only important factor and showed positive influence on H, which is similar to the “Kill-the-Winner” (KtW) model for maintaining high prokaryotic richness (Winter et al., 2010). Besides, VLP may be suppressed under a higher temperature for the adaption of virus to the adverse environment (Tsai et al., 2015b; Stough et al., 2017), which was also reflected in SEM (Figure 5). And there are reports with similar feature that nutrient had positive influence to VLP (Kopylov et al., 2010; Payet and Suttle, 2013). Overall, the established SEM has high credibility, and results showed that virus had obvious influence on phytoplankton diversity.

From Bloom I to Bloom II, the first successional shift was from cyanobacteria-diatom (*Microcystis* sp. and *M. granulata* var. *angustissima* codominance) to cyanobacteria (*Microcystis* sp. dominance) blooms, which can be described as the succession of different phyla. The roles of viral infection may include photosynthetic eukaryotic community suppression and cyanobacterial enhancement, and they were both likely to be work positively for the first succession and helped the success of cyanobacterial bloom.

In our speculation that the suppression of photosynthetic eukaryotic community in the first succession, the VLP was higher in Bloom I (Figure 4A) and also exhibited significant positive relationships with eukaryotic algal species (Figures 6, 7), which showed the potential control of eukaryotic community. Kopylov et al. (2010) found that mesoeutrophic reservoir have higher viral-induced mortality than mesotrophic one. In our study, water with high nutrient levels in Bloom I may contribute to result of the higher VLP. In Lake Taihu, Pound et al. (2020) also observed that the viral lysis may suppresses the eukaryotic community during the *Microcystis* bloom and regarded that the virus-mediated transition from diatoms to cyanobacteria could be explained within the context of a modified version of the KtW model. However, different from the relatively stable hydrological conditions in Lake Taihu, the hydrological conditions in Xiangxi Bay were much more complex due to the reservoir operation (Xu et al., 2011). These hydrodynamic changes shaped the abiotic environments significantly and affected the algal bloom in Xiangxi Bay (Yang et al., 2018). Unavoidably, the hydrodynamic conditions (including tide, thermal stratification, flow velocity changes, and water vertical mixing) can strongly affect the virus-host interactions (Auguet et al., 2005; Barros et al., 2010; Säwström and Pollard, 2012; Chen et al., 2019). Although hydrodynamic conditions in Xiangxi Bay are susceptible to human manipulation and may affect viral infection, there was still obvious viral lysis suppression on eukaryotic algal community in Xiangxi Bay, which is similar to the role of viruses in Lake Taihu (Pound et al., 2020). The summer hydrodynamic conditions in Xiangxi Bay (including stable stratification, low surface mixing layer depth, and velocity) that created similar with shallow lakes may contributed to this result (Zhao et al., 2012; Yang et al., 2018). However, it is regrettable that potential effect of hydrodynamic changes on the bloom dynamics cannot be discussed due to the lack of monitoring data. In addition, silicon limitation has been found to facilitate the viral infection and mortality of marine diatoms in marine environments (Kranzler et al., 2019). And silicate concentration was significantly reduced in the first bloom succession (Table 1), which may also act as the constraining nutrient that causes the collapse of diatoms and provides opportunities for the later cyanobacterial bloom.

As for cyanobacterial enhancement, there was an increasing abundance of *Microcystis* sp. during the first succession (Table 1) and the highest FLP in *Microcystis* bloom (Figure 4B). The infection characteristic of FLP may also have influenced the phytoplankton composition. Specifically, the Shannon diversity index of the phytoplankton community decreased with increasing FLP (Figure 6). The second component in the PCoA, which captured the second-most amount of variance in the phytoplankton communities (Figure 6), showed a positive correlation with FLP. Previous studies have shown that *Microcystis* can resist viral lysis through high number of restriction modification systems (Zhao et al., 2018), and may also minimize losses by forming a lysogenic state (Stough et al., 2017). Knowles et al. (2016) found that the viral densities were more consistent with temperate than lytic life cycles with increasing microbial abundance and growth rates, which was the infection strategy of the PtW model. The potential for *Microcystis* to resist viral lysis and increase dominance by forming a lysogenic state could be another important factor that allows for cyanobacterial dominance and the first succession.

From Bloom II to Bloom III, the second succession was the bloom of different cyanobacterial genera (*Microcystis* sp. dominance to *Microcystis* sp. and *A. circinalis* codominance). VLB, which has significant contribution in RDA model (Table 2), showed a positive relationship with different species (e.g., *A. circinalis* and *A. azotica*; Figure 7). Nutrients released by viral lysis of heterotrophic bacteria may help the second succession and contribute to the continued success of cyanobacterial bloom. And indeed, there exist a decline of bacterioplankton along the succession (Supplementary Table S1) and an increase of PO_4_^3−^ in Bloom III (Table 1). In Bloom II and III, TN/TP ratios were 16.1 and 12.6, respectively. And there was the lowest TN in Bloom III (Table 1). Tian et al. (2012) also found the decrease of nitrate in summer bloom of *Anabaena* in Xiangxi Bay and N limitation was considered as an important cause. Besides, the heterocytes existed in the morphological features of *Anabaena* in Bloom III during the phytoplankton species identification, which is a typical characteristic for nitrogen fixation (Golden and Yoon, 1998). And there was a high concentration of TN in Bloom I, which *Anabaena* was not dominant during this period. Hence, there was high possibility of N limitation in Bloom III, which may cause the replacement of non-N_2_-fixing *Microcystis* sp. by N_2_-fixing *A. circinalis.* Previous studies showed a competitive advantage of *Microcystis* at low P concentrations because of its ability to rapidly uptake and store inorganic P, which also caused P deficiency to other coexisting phytoplankton species in Bloom II (Wan et al., 2019). During the second succession, the PO_4_^3−^ concentration in Xiangxi Bay was still low, but an increase in Bloom III (Table 1), which might be caused by the increase of the VLB, and may help the growth of N_2_-fixing species. Importantly, N_2_ fixation, a metabolically expensive process, is controlled by P availability (Wang et al., 2018). Vanderhoef et al. (1974) found the increased algal growth and nitrogen fixation are correlated with higher phosphate concentrations. Weinbauer et al. (2011) found that the abundant regenerative nutrients required for *Synechococcus* growth were adequately provided due to the viral lysis of heterotrophic bacteria. Tsai (2020) emphasized the importance of the viral shunt of bacteria and quantified the P nutrient released by the viral lysis to be approximately 597.6 ng P L^−1^ d^−1^ in coastal waters. From Bloom II to Bloom III, the VLB followed a rising trend, the average size increasing from 0.211 to 0.312 d^−1^ (Figure 4), which may also contribute to the increase of PO_4_^3−^ concentration from 0.001 mg/L in Bloom II to 0.004 mg/L in Bloom III (Table 1) and finally help the growth of N_2_-fixing species.

This study found that the variation of phytoplankton communities during summer bloom successions was closely associated with the changes in abiotic environmental factors (i.e., T, TN, and PO_4_^3−^) and viral factors (i.e., VA, VLP, FLP, and VLB; Table 2; Figures 6, 7). Hierarchical partitioning method has been widely used to estimate the individual importance of each explanatory variable (Li et al., 2022; Pecuchet et al., 2022). Based on the hierarchical partitioning analysis, the viral variables still have a marked effect on the dynamics of phytoplankton community, although the environmental attributes were the major factors (Table 2). Studies on the estimate of the viral lysis in bloom succession are scarce, so the importance of viruses compared to other factors remain uncertain. However, our results quantified the size of the viral infection characteristics of phytoplankton and bacterioplankton, and showed that viruses may play an important role in summer bloom succession (Figures 5–7). Moreover, our findings were consistent with other studies which showed that viruses were important in bloom control (Jacquet et al., 2002; Du et al., 2020). In general, viruses may play vital roles in phytoplankton community regulation, but these roles may be ignored. And our results highlighted the need for understanding viral infection dynamics in realistic environmental contexts to better predict their biogeochemical consequences (Mojica et al., 2016; Zimmerman et al., 2020).

## 5. Conclusion

The roles of viruses in cyanobacterial blooms and taxa succession have yet to be well elucidated in aquatic ecosystems, including the world largest hydropower reservoir, TGR. In this study, viral infection characteristics monitoring during the summer bloom successions was conducted to determine the effects of viruses on cyanobacterial bloom in Xiangxi Bay of TGR. The main conclusions were drawn as follows:

1. Viruses may promote the cyanobacterial blooms and work by multiple and complex ways, including the enhanced lysis of eukaryotic community, the increase of lysogeny in cyanobacteria, and the nutrients supplied from the lysis of bacterioplankton.
2. Multiple potential roles of viruses were found during the bloom succession, highlighting the complexity of viral regulation in helping the summer cyanobacteria bloom success in Xiangxi Bay. Neither the KtW model nor the PtW model can fully explain the roles of viruses in summer bloom succession of Xiangxi Bay. The theoretical framework of virus-host interaction may need further modification.

## Data availability statement

The original contributions presented in the study are included in the article/Supplementary material, further inquiries can be directed to the corresponding author.

## Author contributions

ML, YZ, and SY conceived and designed the study. YJ and JG performed the analyses. YJ provided the additional sample material. ML, YZ, and WX contributed to the project administration. KP analyzed the results and wrote the manuscript, which was edited and approved by all authors. All authors contributed to the article and approved the submitted version.

## Funding

This work was supported by the National Natural Science Foundation of China (No. 51579092) and the National Key Basic Research Program of China (No. 2016YFC0401702).

## Conflict of interest

The authors declare that the research was conducted in the absence of any commercial or financial relationships that could be construed as a potential conflict of interest.

## Publisher’s note

All claims expressed in this article are solely those of the authors and do not necessarily represent those of their affiliated organizations, or those of the publisher, the editors and the reviewers. Any product that may be evaluated in this article, or claim that may be made by its manufacturer, is not guaranteed or endorsed by the publisher.
